# Monkeypox Clade Ib virus introduction into Burundi: first findings, July to mid-August 2024

**DOI:** 10.2807/1560-7917.ES.2024.29.42.2400666

**Published:** 2024-10-17

**Authors:** Néhémie Nzoyikorera, Cassien Nduwimana, Leonard Schuele, David F Nieuwenhuijse, Marion Koopmans, Saria Otani, Frank M Aarestrup, Théogène Ihorimbere, Denis Niyomwungere, Armstrong Ndihokubwayo, Idrissa Diawara, Alexis Niyomwungere, Dionis Nizigiyimana, Marie Noelle Uwineza, Bas B Oude Munnink, Joseph Nyandwi

**Affiliations:** 1 National Reference Laboratory, National Public Health Institute, Bujumbura, Burundi; 2 Department of Viroscience, Erasmus University Medical Center, Rotterdam, the Netherlands; 3 Research Group for Genomic Epidemiology, National Food Institute, Technical University of Denmark, Kgs. Lyngby, Denmark; 4 Laboratoire d'immunologie clinique, Inflammation et Allergie (LICIA), Faculté de Médecine et de Pharmacie de Casablanca, Université Hassan II, Casablanca, Morocco; 5 Mohammed VI University of Sciences and Health, Mohammed VI Higher Institute of Biosciences and Biotechnologies (UM6SS), Casablanca, Morocco; 6 Infectious Diseases Research Unit, Mohammed VI Center for Research & Innovation (CM6RI), Rabat, Morocco; 7 Organisation mondiale de la Santé, Burundi; 8 National Public Health Institute, Ministry of Public Health and the Fight against AIDS, Bujumbura, Burundi; 9 Faculté de Médecine, Université du Burundi, Bujumbura, Burundi; * These authors contributed equally to this work and share the last authorship.

**Keywords:** Mpox, Clade Ib, WGS, Epidemiology, Burundi

## Abstract

We describe cases with monkeypox virus (MPXV) Clade Ib in Burundi from their first detection in July until 20 August 2024. Testing 442 people with vesicular lesions confirmed 170 cases (98 male; 72 female), 82 (48%) being < 15 years old. Differential diagnosis of the first 30 individuals testing MPXV negative revealed chickenpox in 20. Cases occurred in 26 of 49 Burundi health districts, but mostly in Bujumbura Nord (88/170; 67%). Case-derived MPXV genetic sequences from Burundi and South-Kivu (Democratic Republic of the Congo), clustered together in phylogenetic analysis.

From May 2023, a sharp increase of mpox cases due to MPXV Clade I was observed across the Democratic Republic of Congo (DRC), with cases occurring in areas where MPXV had not prior been detected [1]. Investigations indicated ongoing virus evolution and the co-circulation of several different Clade I MPXV sub-lineages in DRC [2,3]. Subsequent epidemiological, sequencing, and phylogenetic analyses revealed that MPXV of Clade Ib was spreading geographically within the DRC and cases were detected in other countries like Burundi, India, Kenya, Rwanda, Sweden, Tanzania, Thailand and Uganda [4]. The World Health Organization declared mpox a public health emergency of international concern on 14 August 2024 [5]. Here we document the characteristics of laboratory-confirmed mpox cases caused by Clade Ib in Burundi and their geographic distribution during the first month of the outbreak in the country, as well as phylogenetically analyse viral whole genome sequences affecting local cases.

## Monkeypox virus Clade Ib outbreak in Burundi

**Figure 1:**
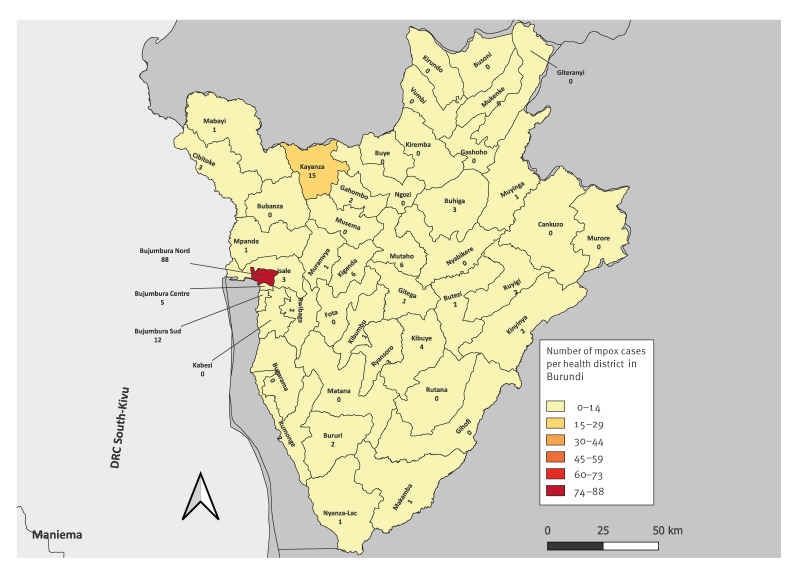
Distribution of laboratory-confirmed mpox cases in different health districts of Burundi up to 20 August 2024 (n = 170 cases)

MPXV reached Burundi by 25 July 2024, when the first three cases were reported in the country [6]. The three cases were detected in two adjacent health districts, Bujumbura Nord (in Bujumbura City) and Isale health districts (Figure 1). These health districts are geographically located in western Burundi, bordering the DRC. Despite the implementation of public health and social measures, transmission continued, and in the following days, more mpox cases were detected in different health districts across the country.

The numbers indicate the number of mpox cases confirmed by laboratory analysis, up to 20 August 2024.

In Burundi, patients presenting to hospital with vesicular lesions are tested for MPXV infection via analysis of respective swab samples of the lesion(s). When a patient’s sample tests positive by PCR for MPXV, the patient is hospitalised for treatment and isolation. When it is negative and in absence of another condition requiring hospitalisation, the patient is released. Mpox diagnosis is performed centrally at the National Reference Laboratory of Burundi, located in Bujumbura Mairie province, on vesicular swabs collected from all patients with vesicular lesions using a general real-time (RT)-PCR for MPXV detection [7]. This is followed by the Clade Ib specific RT-PCR using the TaqMan Fast Advanced Master Mix for RT-PCR as prior described [2].

The demographic and clinical data of people tested for MPXV are collected using a national standardised data collection form designed for mpox. For the current study, we retrieved these data for all laboratory-confirmed mpox cases, who are further referred to as confirmed mpox cases. Data included sex (male/female), place of residence, date of sample collection and age in year and month. Severity of disease presentation is not recorded.

Up to 20 August 2024, 170 mpox cases have been confirmed by RT-PCR in Burundi, accounting for 38.5% of all the patients tested (n = 442) during that period. All confirmed cases were caused by MPXV of Clade Ib. The western part of Burundi was most affected by the mpox outbreak with 61.8% of cases detected (105/170) in Bujumbura Mairie province (comprising Bujumbura health districts Nord, Centre and Sud; Figure 1), followed by Kayanza health district with 8.8% of cases (15/170), and Mutaho and Kiganda health districts with 3.5% (6/170) of cases each. Overall, the mpox outbreak spread to many parts of the country affecting 26 of the 49 health districts (Figure 1). All the positive cases were hospitalised and no death has been reported.

## Demographic characteristics of confirmed mpox cases and differential diagnosis

**Figure 2:**
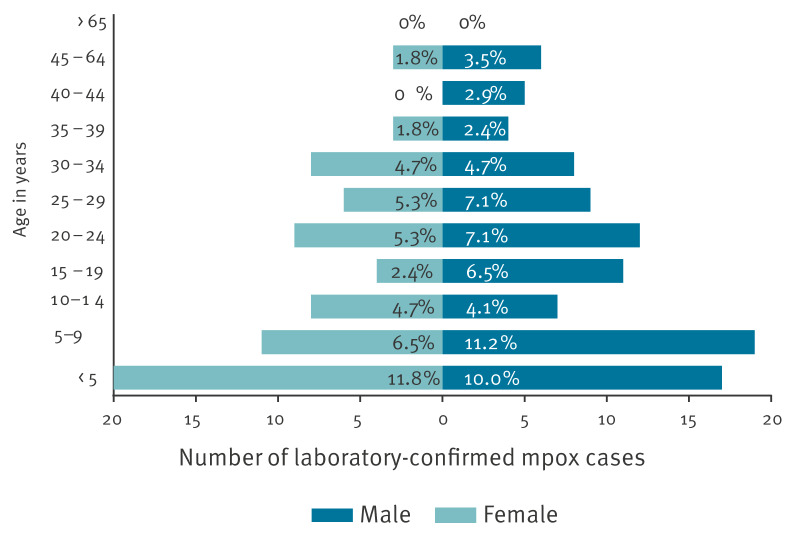
Laboratory-confirmed mpox cases disaggregated by age and sex, Burundi up to 20 August 2024 (n = 170 cases)

**Figure 3:**
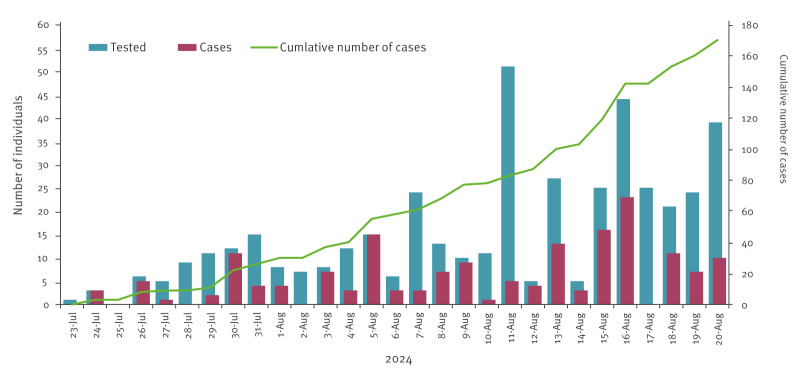
Distribution of laboratory-confirmed mpox cases in Burundi up to 20 August 2024 (n = 170 cases)

The age of the confirmed mpox cases ranged between 2 months and 65 years (mean: 17.05 years). Around half of the cases (82/170; 48%) were children under 15 years old and 30% (51/170) were between 15 and 29 years old. In total, 42.4% (72/170) of the cases were female and 57.6% (98/170) male (Figure 2). A peak of cases was observed on 16 August 2024 with 23 cases confirmed on that day (Figure 3). Furthermore, the number of health districts affected increased progressively with up to 26 health districts affected up to 20 August 2024.

The green line shows the cumulative number of mpox cases during the first month of the outbreak while the bar charts show the number of patients tested (blue) and the number of laboratory-confirmed mpox cases (red) per day.

Differential diagnostics using AmpliSens VZV-FL and AmpliSens HSV I,II-FL RT-PCR kits were conducted on the first set of MPXV negative cases. Of 30 samples, 20 were positive for varicella zoster virus (VZV) while four samples were positive for herpes simplex virus (HSV)-1,2. Chickenpox, which is caused by VZV, predominantly affected children between 1 and 17 years of age.

## Whole genome sequencing and phylogenetic analysis

**Figure 4:**
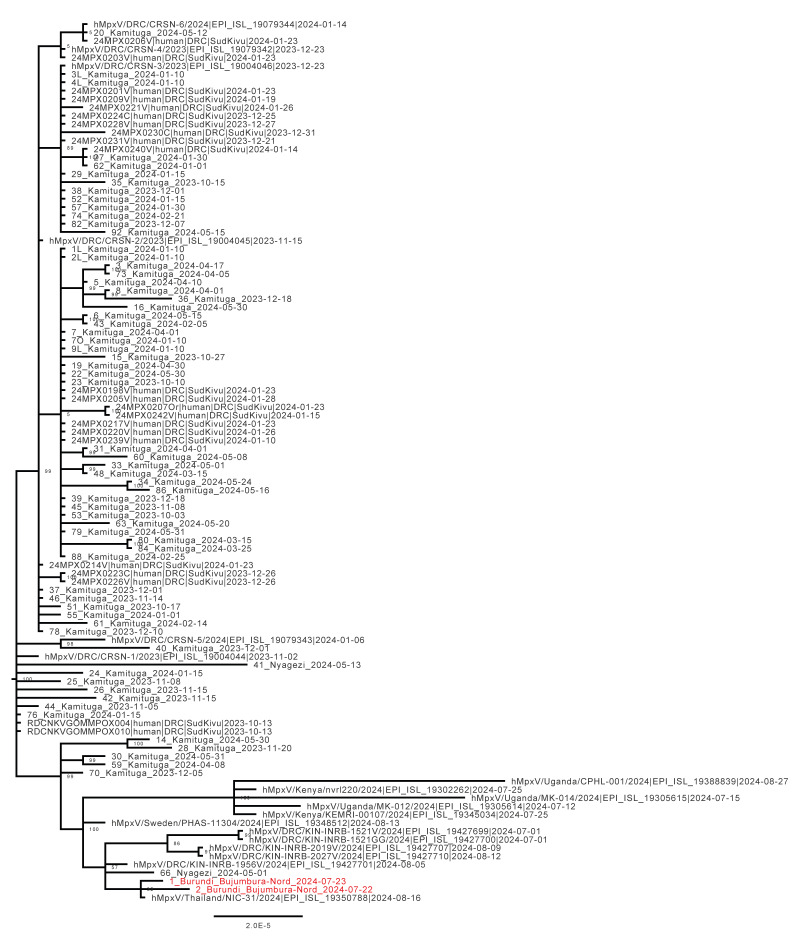
Phylogenetic analysis of whole genome sequences from MPXV derived from mpox cases in Burundi along with all publicly available complete Clade Ib MPXV sequences

Samples from the first two confirmed mpox cases in the most affected health district (Bujumbura Nord) were submitted for whole genome sequencing and analysis, using a prior described amplicon-based sequencing approach [8]. A multiple sequence alignment was performed using Mafft (https://mafft.cbrc.jp/alignment/server/) after which we performed a phylogenetic analysis using IQ-Tree2 [9] with all complete (> 85% coverage over the genome) publicly available Clade Ib MPXV sequences on National Center for Biotechnology Information (NCBI) and on GISAID using the ultrafast bootstrapping option. This revealed that sequences of the MPXV affecting the cases in Burundi clustered with sequences prior detected in the health zones of Kamituga and Kamanyola in South Kivu, DRC and some sequences of MPXV that further spread internationally (Figure 4). The sequences recovered in this work have been deposited in GISAID (see data availability).

The sequences derived from this study are shown in red. Numbers at the nodes represent bootstrap values. The scale bar represents the number of substitutions per site. All publicly available sequences with greater than 85% genome coverage at the time of analysis were included to derive the phylogenetic tree. More details on the GISAID sequences used for the tree are included in Supplementary Table 1.

## Discussion

In this study, we describe the number of cases and their age distribution in the first month of the mpox Clade Ib outbreak in Burundi and provide the first two genetic sequences of the MPXV causing this outbreak. The emergence of mpox and the increasing number of cases in Burundi came in the context of a multi-country mpox outbreak, suggesting an ongoing spread of MPXV [10,11]. The movement of people between South Kivu (DRC) and Bujumbura Mairie province (Burundi) is suggested to have contributed to the spread of mpox from the DRC, where MPXV of Clade Ib had been described before. The mpox outbreak spread across many health districts of Burundi but a higher prevalence of cases was observed in the western part, mainly the Bujumbura Mairie province, the most densely populated city in Burundi. The population density in Bujumbura Mairie could be the reason why most cases are detected there, next to the province being in close geographical proximity to South Kivu. This epidemiological situation strongly suggests continued human-to-human transmission of MPXV driving this outbreak as described elsewhere [12,13].

Our study revealed that almost half of confirmed cases found in Burundi were children under 15 years old and that 30% were between 15 and 29 years old. These proportions seem to differ from the age profiles found in an investigation in DRC among mpox cases caused by Clade Ib [13], where the age group under 15 years only comprised 14.8% (16/108) of cases and the age group between 15 and 30 years comprised 67% (73/108) of cases. In the DRC investigation, a potential role for sexual transmission was suggested [13]. As our questionnaire did not include questions on potential risk exposures, we cannot draw conclusions on the modes of transmission. For children under 15 years of age, however, it is striking that they constitute the most affected age group of notified cases in Burundi. Possible explanations are that mpox is under-reported in young adults in Burundi, making them appear less affected than in DRC, where mpox in this group is more commonly found, or an increase of non-sexual human-to-human transmission modes involved in the outbreak occurring in Burundi. This question needs to be urgently addressed in future studies. The high prevalence of chickenpox in MPXV negative cases also shows the importance of diagnostic capacity in addition to clinical case reporting. This capacity should include differential diagnostics, beyond MPXV detection.

Following the declaration of the mpox outbreak in Burundi by the Minister of Health on 25 July 2024, public health and social measures have been set to contain the epidemic. These measures included frequent hand washing with alcohol-based solutions or water and soap. These measures proved to be effective in reducing the transmission of respiratory and contact-transmitted diseases such as influenza and COVID-19. However, in the current outbreak, the number of mpox cases kept increasing, suggesting their limitations or that people complied less to them and that other measures are urgently needed to slow the further spread of mpox Clade Ib. Vaccination against MPXV has not yet introduced in Burundi because it has not yet been introduced in the national immunisation strategy.

Our study has some limitations. There may have been under-reporting of cases in the community. The contact tracing of confirmed mpox cases or their parents was not done during the data collection process. Consequently, we were not able to assess the role played by sexual activities in the spread of mpox in Burundi. Moreover, the demographic and geographical information of those tested was not considered, thus the group of people tested for MPXV is not compared to the group of confirmed mpox cases in this respect. 

## Conclusion 

This study provides a description of the early epidemiology of the mpox outbreak in Burundi and the clade involved. The outbreak, caused only by the Clade Ib first detected in South Kivu in the DRC, has expanded to different health districts of Burundi with a hotspot in Bujumbura health districts. We observed that the public health measures put in place since the beginning of the outbreak have not reduced the outbreak transmission, showing the importance of further intensified public health interventions for this evolving outbreak to prevent cross-border spread and break the transmission chain within the country. The difference in impact on children aged under 15 years in DRC and Burundi stresses the need for continued vigilance including virological monitoring and further investigation. In addition, surveillance at the genomic level and community engagement are critical for an effective response to that emerging public health threat.

## ^†^Authors’ correction

Upon publication, the name of author Marie Noelle Uwineza was spelled incorrectly. This was corrected on 18 October 2024 at the request of the authors.

## Supplementary Data

468000 Supplementary Table 1
