# Authors’ correction for Euro Surveill. 2024;29(42)

**DOI:** 10.2807/1560-7917.ES.2024.29.43.241018c

**Published:** 2024-10-24

**Authors:** Eurosurveillance editorial team

**Affiliations:** 1 European Centre for Disease Prevention and Control (ECDC), Stockholm, Sweden

In the article entitled ‘Monkeypox Clade Ib virus introduction into Burundi: first findings, July to mid-August 2024’ by Nzoyikorera et al., published on 17 October 2024, the name of author Marie Noelle Uwineza was spelled incorrectly. This was corrected on 18 October 2024 at the request of the authors.

