# Supplementary material for: Monkeypox Clade Ib virus introduction into Burundi: first findings, July to mid-August 2024
Source: Euro Surveill. 2024 Oct 17;29(42):2400666. doi: 10.2807/1560-7917.ES.2024.29.42.2400666 (PMC11487920; doi:10.2807/1560-7917.ES.2024.29.42.2400666)
Supplement: Supplementary Table 1 [file 2400666_SupplementaryTable1.pdf]

This supplementary material is hosted by *Eurosurveillance* as supporting information alongside the article "Monkeypox Clade Ib virus introduction into Burundi: first findings, July to mid-August 2024" on behalf of the authors, who remain responsible for the accuracy and appropriateness of the content. The same standards for ethics, copyright, attributions and permissions as for the article apply. Supplements are not edited by *Eurosurveillance* and the journal is not responsible for the maintenance of any links or email addresses provided therein.

Monkeypox Clade Ib virus introduction into Burundi: first findings, July to mid-August 2024

Supplementary Table 1

We gratefully acknowledge the following Authors from the Originating laboratories responsible for obtaining the specimens, as well as the Submitting laboratories where the genome data were generated and shared via GISAID, on which this research is based.

All Submitters of data may be contacted directly via www.gisaid.org

Authors are sorted alphabetically.

Accession ID	Originating Laboratory	Submitting Laboratory	Authors
EPI_ISL_18886301	Reseau lab Bukavu-Kamituga DPS/ Sud -Kivu	Reseau lab Bukavu-Kamituga DPS/ Sud-Kivu	Leandre Murhula Masirika, Jean Claude Udahemuka, Leonard Schuele, Pacifique Ndishimye, Saria Otani ,Justin Bengehya Mbiribindi, Jean M. Marekani, Léandre Mutimbwa Mambo, Marjan Boter, David F. Nieuwenhuijse,Ernest Balyahamwabo Kalalizi,Trudie Lang, Jean Pierre Musabyimana, Frank M. Aarestrup, Marion Koopmans, Bas B. Oude Munnink, Freddy Belesi Siangoli
EPI_ISL_18886467	Reseau Lab Bukavu -Kamituga DPS/Sud-Kivu	Reseau Lab Bukavu-Kamituga DPS /Sud-Kivu	Leandre Murhula Masirika, Jean Claude Udahemuka, Leonard Schuele, Pacifique Ndishimye, Saria Otani ,Justin Bengehya Mbiribindi, Jean M. Marekani, Léandre Mutimbwa Mambo, Marjan Boter, David F. Nieuwenhuijse,Ernest Balyahamwabo Kalalizi,Trudie Lang, Jean Pierre Musabyimana, Frank M. Aarestrup, Marion Koopmans, Bas B. Oude Munnink, Freddy Belesi Siangoli
EPI_ISL_18886588	Reseau lab Bukavu-Kamituga DPS/Sud-Kivu	Reseau lab Bukavu -Kamituga DPS /Sud -Kivu	Leandre Murhula Masirika, Jean Claude Udahemuka, Leonard Schuele, Pacifique Ndishimye, Saria Otani ,Justin Bengehya Mbiribindi, Jean M. Marekani, Léandre Mutimbwa Mambo, Marjan Boter, David F. Nieuwenhuijse,Ernest Balyahamwabo Kalalizi,Trudie Lang, Jean Pierre Musabyimana, Frank M. Aarestrup, Marion Koopmans, Bas B. Oude Munnink, Freddy Belesi Siangoli
EPI_ISL_18886634	Reseau Lab Bukavu-Kamituga DPS/Sud-Kivu	Reseau Lab Bukavu-Kamituga DPS/Sud Kivu	Leandre Murhula Masirika, Jean Claude Udahemuka, Leonard Schuele, Pacifique Ndishimye, Saria Otani ,Justin Bengehya Mbiribindi, Jean M. Marekani, Léandre Mutimbwa Mambo, Marjan Boter, David F. Nieuwenhuijse,Ernest Balyahamwabo Kalalizi,Trudie Lang, Jean Pierre Musabyimana, Frank M. Aarestrup, Marion Koopmans, Bas B. Oude Munnink, Freddy Belesi Siangoli
EPI_ISL_18886635	Reseau Lab Bukavu-Kamituga DPS/Sud Kivu	Reseau Lab Bukavu-Kamituga DPS/Sud Kivu	Leandre Murhula Masirika, Jean Claude Udahemuka, Leonard Schuele, Pacifique Ndishimye, Saria Otani ,Justin Bengehya Mbiribindi, Jean M. Marekani, Léandre Mutimbwa Mambo, Marjan Boter, David F. Nieuwenhuijse,Ernest Balyahamwabo Kalalizi,Trudie Lang, Jean Pierre Musabyimana, Frank M. Aarestrup, Marion Koopmans, Bas B. Oude Munnink, Freddy Belesi Siangoli
EPI_ISL_18886639	Reseau Lab Bukavu-Kamituga DPS/ SUD-Kivu	Reseau Lab Bukavu-Kamituga DPS/ Sud-Kivu	Leandre Murhula Masirika, Jean Claude Udahemuka, Leonard Schuele, Pacifique Ndishimye, Saria Otani ,Justin Bengehya Mbiribindi, Jean M. Marekani, Léandre Mutimbwa Mambo, Marjan Boter, David F. Nieuwenhuijse,Ernest Balyahamwabo Kalalizi,Trudie Lang, Jean Pierre Musabyimana, Frank M. Aarestrup, Marion Koopmans, Bas B. Oude Munnink, Freddy Belesi Siangoli
EPI_ISL_19004044	Centre de Recherche en Sciences Naturelles de Lwiro	Centre de Recherche en Sciences Naturelles de Lwiro	Leandre Murhula Masirika, Jean Claude Udahemuka, Pacifique Ndishimye, Gustavo Sganzerla Martinez, Patricia Kelvin, Maliyamungu Bubala Nadine, Bilembo Kitwanda Steeven, Franklin Kumbana Mweshi, Léandre Mutimbwa Mambo, Bas B. Oude Munnink, Justin Bengehya Mbiribindi, Freddy Belesi Siangoli, Trudie Lang, Jean M. Malekani, Frank M. Aarestrup, Marion Koopmans, Leonard Schuele, Jean Pierre Musabyimana, Brigitte Umutoni, Ali Toloue, Benjamin Hewins, Mansi Dutt, Anuj Kumar, Alyson A. Kelvin, Jean-Paul Kabemba Lukusa, Christian Gortazar, David J Kelvin, Luis Flores
EPI_ISL_19004045, EPI_ISL_19004046	Centre de Recherche en Sciences Naturelles de Lwiro	Centre de Recherche en Sciences Naturelles de Lwiro	Leandre Murhula Masirika , Jean Claude Udahemuka, Pacifique Ndishimye, Gustavo Sganzerla Martinez, Patricia Kelvin, Maliyamungu Bubala Nadine, Bilembo Kitwanda Steeven, Franklin Kumbana Mweshi, Léandre Mutimbwa Mambo, Bas B. Oude Munnink, Justin Bengehya Mbiribindi, Freddy Belesi Siangoli, Trudie Lang, Jean M. Malekani, Frank M. Aarestrup, Marion Koopmans, Leonard Schuele, Jean Pierre Musabyimana, Brigitte Umutoni, Ali Toloue, Benjamin Hewins, Mansi Dutt, Anuj Kumar, Alyson A. Kelvin, Jean-Paul Kabemba Lukusa, Christian Gortazar, David J Kelvin, Luis Flores
EPI_ISL_19079342, EPI_ISL_19079343, EPI_ISL_19079344	Centre de Recherche en Sciences Naturelles de Lwiro (CRSN Lwiro)	Centre de Recherche en Sciences Naturelles de Lwiro (CRSN Lwiro)	Leandre M Masirika, Anuj Kumar, Mansi Dutt, Ali Toloue Ostadgavahi, Benjamin Hewins, Maliyamungu B Nadine, Bilembo K Steeven, Franklin K Mweshi, Léandre M Mambo, Justin B Mbiribindi, Freddy B Siangoli, Alyson A Kelvin, Jean Claude Udahemuka, Patricia Kelvin, Luis Flores, David J Kelvin, Gustavo Sganzerla Martinez
EPI_ISL_19093791, EPI_ISL_19093792, EPI_ISL_19093794, EPI_ISL_19093796, EPI_ISL_19093797, EPI_ISL_19093800, EPI_ISL_19093803, EPI_ISL_19093808, EPI_ISL_19093809, EPI_ISL_19093810, EPI_ISL_19093811, EPI_ISL_19093812, EPI_ISL_19093814, EPI_ISL_19093815, EPI_ISL_19093816, EPI_ISL_19093817, EPI_ISL_19093827, EPI_ISL_19093830, EPI_ISL_19093831, EPI_ISL_19093833, EPI_ISL_19093834, EPI_ISL_19093835			
see above	Pathogen Genomic Laboratory, Institut National de Recherche Biomedicale	Pathogen Genomic Laboratory, Institut National de Recherche Biomedicale	Vakaniaki,E.H., Kacita,C., Kinganda - Lusamaki,E., O'Toole,A., Wawina -Bokalanga,T., Mukadi - Bamuleka,D., Amuri,A.A., Parker,E., Muswamba-Kayembe,P.-C., Makangara - Cigolo,J.-C., Mulopo - Mukanya,N., Pukuta - Simbu,E., Mujula,Y., Nundu,S.S., Akil - Bandali,P., Kavunga,H., Lushima,R.S., Vercauteren,K., Sam-Agudu,N.A., Mills,E.J., Tshiani - Mbaya,O., Hoff,N., Rimoin,A.W., Hensley,L.E., Kundrachuk,J., Ayouba,A., Peeters,M., Delaporte,E., Nachega,J.B., Ahuka - Mundeke,S., Muyembe - Tamfum,J.-J., Rambaut,A., Liesenborghs,L. and Mbala - Kingebeni,P.
EPI_ISL_19302262	National Public Health Laboratory - NVRL	Kenya Medical Research Institute (KEMRI) / Walter Reed Army Institute of Research - Africa	Gathii Kimita, Joseph K. Kaingu, George O. Awinda, Allan P. Lemtudo, Esther A. Omuseni, Josphat N. Nyataya, Beth K. Mutai, John N. Waitumbi
EPI_ISL_19305614, EPI_ISL_19305615	MRC/UVRI & LSHTM Uganda Research Unit, Uganda Virus Research Institute	MRC/UVRI & LSHTM Uganda Research Unit, Uganda Virus Research Institute	Nicholas Bbosa, Stella E. Nabirye, Hamidah S. Namagembe, Ronald Kiiza, Alfred Ssekagiri, Mary Munyagwa, Arafat Bwambale, Stephen Bagonza, Henry Kyobe Bosa, Mary Rodgers, Francisco Averhoff, Michael Berg, Robert Downing, Gavin Cloherty, Julius Lutwama, Pontiano Kaleebu, Deogratius Ssemwanga
EPI_ISL_19345034	Arbovirology/Viral Haemorrhagic Fever Lab, Kenya Medical Research Institute (KEMRI)	Arbovirology/Viral Haemorrhagic Fever Lab, Kenya Medical Research Institute (KEMRI)	Langat,S., Nyunja,A., Pilarowski,G., Okunga,E., Ofula,V., Oluniyi,P., Koskei,E., Koka,H., Owaka,S., Chepkorir,E., Lutomiah,J., Langat,D., Khamadi,S. and Limbaso,K.
EPI_ISL_19348512	The Public Health Agency of Sweden	The Public Health Agency of Sweden	Oskar Karlsson Lindsjö, Maria Lind Karlberg, Klara Sondén
EPI_ISL_19350788	Hospital in Bangkok	Department of Medical Sciences, National Institute of Health	Pilailuk Okada; Siripaporn Phuygun; Nuttida Thongpramul, Titichaya Mebuathong, Pakorn Piromtong, Thanutsapa Thanadachakul, Thitipong Yingyong, Archawin Rojanawiwat, Ballang Uppapong, Yongyos Thammavuth
EPI_ISL_19357138, EPI_ISL_19357623, EPI_ISL_19357654	Hôpital Général de Référence de Kamituga	Hôpital Général de Référence de Kamituga	Leandre Murhula Masirika, David F. Nieuwenhuijse, Leonard Schuele, Pacifique Ndishimye, Jean Claude Udahemuka, Justin Bengehya Mbiribindi, Christian Cortazar, Frank M. Aarestrup, Freddy Belesi Siangoli, Bas Oude Munnink, Trudie Lang, Marion Koopmans
EPI_ISL_19361815, EPI_ISL_19361896, EPI_ISL_19361897, EPI_ISL_19363685, EPI_ISL_19363686, EPI_ISL_19364035	Kamituga General Reference Hospital	Kamituga General Reference Hospital	Leandre Murhula Masirika, David F. Nieuwenhuijse, Leonard Schuele, Pacifique Ndishimye, Jean Claude Udahemuka, Justin Bengehya Mbiribindi, Christian Cortazar, Frank M. Aarestrup, Freddy Belesi Siangoli, Bas Oude Munnink, Trudie Lang, Marion Koopmans
EPI_ISL_19364149, EPI_ISL_19364369, EPI_ISL_19364511, EPI_ISL_19364948, EPI_ISL_19365441, EPI_ISL_19365466, EPI_ISL_19365506, EPI_ISL_19365507, EPI_ISL_19365542, EPI_ISL_19365543, EPI_ISL_19365544, EPI_ISL_19365545, EPI_ISL_19366314			
see above	Kamituga General Reference Hospital	Kamituga General Reference Hospital	Leandre Murhula Masirika, David F. Nieuwenhuijse, Leonard Schuele, Pacifique Ndishimye, Jean Claude Udahemuka, Justin Bengehya Mbiribindi, Christian Cortazar, Frank M. Aarestrup1, Freddy Belesi Siangoli, Bas Oude Munnink, Trudie Lang, Marion Koopmans
EPI_ISL_19366744	Thai Red Cross Emerging Infectious Diseases Clinical Center, King Chulalongkorn Memorial Hospital	Thai Red Cross Emerging Infectious Diseases Clinical Center, King Chulalongkorn Memorial Hospital	Ampoot,W., Rattanatumhi,K., Ruchisrisarod,C., Supataragul,A., Saraya,A.W., Wacharapluesadee,S., Avihingsanon,Y. and Putcharoen,O.
EPI_ISL_19367803, EPI_ISL_19381268, EPI_ISL_19382151, EPI_ISL_19382354, EPI_ISL_19382355, EPI_ISL_19382807, EPI_ISL_19382808, EPI_ISL_19382908, EPI_ISL_19382942, EPI_ISL_19382949, EPI_ISL_19382965			
see above	Kamituga General Reference Hospital	Kamituga General Reference Hospital	Leandre Murhula Masirika, David F. Nieuwenhuijse, Leonard Schuele, Pacifique Ndishimye, Jean Claude Udahemuka, Justin Bengehya Mbiribindi, Christian Cortazar, Frank M. Aarestrup1, Freddy Belesi Siangoli, Bas Oude Munnink, Trudie Lang, Marion Koopmans
EPI_ISL_19388839	CPHL Mobile Laboratory-Bwera	CPHL Genomics Core Laboratory	Alisen Ayitewala, Godfrey Pimundu, Tonny Muyigi, Hellen Oundo, Wislon Tenywa, Julius Sseruyange, Godwin Tusabe, Moses Murungi, Stephen Kanyerezi, Eunice Nambozo, Joseph Sekate, Rebecca, Nalwanga, Lydia Namakula, Aloysious Ssemaganda, Isaac Ssewanyana, Susan Nabadda
EPI_ISL_19417681	Institut National de Recherche Biomédical	Spiez Laboratory	Muyeme Tamfum Jean Jacques, Mbala Placide
EPI_ISL_19422968, EPI_ISL_19422969	Kamituga General Reference Hospital	Kamituga General Reference Hospital	Leandre Murhula Masirika, David F. Nieuwenhuijse, Leonard Schuele, Pacifique Ndishimye, Jean Claude Udahemuka, Justin Bengehya Mbiribindi, Christian Cortazar, Frank M. Aarestrup1, Freddy Belesi Siangoli, Bas Oude Munnink, Trudie Lang, Marion Koopmans
EPI_ISL_19424850	Institut National de Recherche Biomédical	Spiez Laboratory	Muyeme Tamfum Jean Jacques, Mbala Placide
EPI_ISL_19427699, EPI_ISL_19427700, EPI_ISL_19427701, EPI_ISL_19427707, EPI_ISL_19427710	Pathogen Genomics Lab, National Institute for Biomedical Research (INRB)	Pathogen Genomics Lab, National Institute for Biomedical Research (INRB)	Tony Wawina-Bokalanga, Prince Akil-Bandali, Eddy Kinganda-Lusamaki, Emmanuel Lokilo, Daan Jansen, Adrienne Amuri-Aziza, Jean-Claude Makangara-Cigolo, Elisabeth Pukuta-Simbu, Rilia Ola-Mpumbe, Mamito Muyembe, Cris Kacita, Princesse Paku-Tshambu, Sifa Kavira Bapitani, Pedro H.L.F. Dantas, Olivier Tshiani-Mbaya, Gradi Luakanda-Ndelemo, Antoine Nkuba-Ndaye, Meris Matondo, Junior Bulabula, Ange Ponga Museme, Pauline Musuamba, Nelson Kashali, Emmanuel Hasivirwe Vakaniaki, Sofonias Tessema, Nicaise Ndembi, Áine O'Toole, Tessa De Block, Christian Ngandu, Nicole A. Hoff, Nicola Low, Lorenzo Subissi, Sydney Merritt, Jean-Jacques Muyembe-Tamfum, Laurens Liesenborghs, Martine Peeters, Eric Delaporte, Jason Kindrachuk, Anne W. Rimoin, Steve Ahuka-Mundeke, Andrew Rambaut, Dieudonné Mwamba, Koen Vercauteren, Placide Mbala-Kingebeni
EPI_ISL_19429464	Kamituga General Reference Hospital /South-Kivu	Kamituga General Reference Hospital /South-Kivu	Leandre Murhula Masirika, David F. Nieuwenhuijse, Leonard Schuele, Pacifique Ndishimye, Jean Claude Udahemuka, Justin Bengehya Mbiribindi, Christian Cortazar, Frank M. Aarestrup1, Freddy Belesi Siangoli, Bas Oude Munnink, Trudie Lang, Marion Koopmans
EPI_ISL_19429465, EPI_ISL_19429466	Kamituga General Reference Hospital/South-Kivu	Kamituga General Reference Hospital/South-Kivu	Leandre Murhula Masirika, David F. Nieuwenhuijse, Leonard Schuele, Pacifique Ndishimye, Jean Claude Udahemuka, Justin Bengehya Mbiribindi, Christian Cortazar, Frank M. Aarestrup1, Freddy Belesi Siangoli, Bas Oude Munnink, Trudie Lang, Marion Koopmans
EPI_ISL_19431759, EPI_ISL_19431760, EPI_ISL_19431761, EPI_ISL_19431762, EPI_ISL_19431763, EPI_ISL_19433147	Kamituga General Reference Hospital/South-Kivu	Kamituga General Reference Hospital /South-Kivu	Leandre Murhula Masirika, David F. Nieuwenhuijse, Leonard Schuele, Pacifique Ndishimye, Jean Claude Udahemuka, Justin Bengehya Mbiribindi, Christian Cortazar, Frank M. Aarestrup1, Freddy Belesi Siangoli, Bas Oude Munnink, Trudie Lang, Marion Koopmans
EPI_ISL_19434036, EPI_ISL_19434037, EPI_ISL_19434038, EPI_ISL_19434063, EPI_ISL_19434064, EPI_ISL_19439694, EPI_ISL_19439695, EPI_ISL_19439696, EPI_ISL_19439697	Kamituga General Reference Hospital	Kamituga General Reference Hospital	Leandre Murhula Masirika, David F. Nieuwenhuijse, Leonard Schuele, Pacifique Ndishimye, Jean Claude Udahemuka, Justin Bengehya Mbiribindi, Christian Cortazar, Frank M. Aarestrup1, Freddy Belesi Siangoli, Bas Oude Munnink, Trudie Lang, Marion Koopmans
EPI_ISL_19440397	The Public Health Agency of Sweden	The Public Health Agency of Sweden	Oskar Karlsson Lindsjö, Maria Lind Karlberg, Klara Sondén
EPI_ISL_19456777	CPHL Mobile Laboratory-Bwera	CPHL Genomics Core	Alisen Ayitewala, Godfrey Pimundu, Tonny Muyigi, Hellen Oundo, Wislon Tenywa, Julius Sseruyange, Godwin Tusabe, Moses Murungi, Stephen Kanyerezi, Eunice Nambozo, Joseph Sekate, Rebecca, Nalwanga, Lydia Namakula, Aloysious Ssemaganda, Isaac Ssewanyana, Susan

		Laboratory	Nabadda
EPI_ISL_19460816, EPI_ISL_19460883, EPI_ISL_19460954, EPI_ISL_19461017, EPI_ISL_19462380, EPI_ISL_19462381	Kamituga General Reference Hospital	Kamituga General Reference Hospital	Leandre Murhula Masirika, David F. Nieuwenhuijse, Leonard Schuele, Pacifique Ndishimye, Jean Claude Udahemuka, Justin Bengehya Mbiribindi, Christian Cortazar, Frank M. Aarestrup1, Freddy Belesi Siangoli, Bas Oude Munnink, Trudie Lang, Marion Koopmans
